# Lead palladium titanate: A room temperature nanoscale multiferroic thin film

**DOI:** 10.1038/s41598-020-59961-w

**Published:** 2020-02-19

**Authors:** K. K. Mishra, Alvaro A. Instan, Shalini Kumari, J. F. Scott, Ram S. Katiyar

**Affiliations:** 1 Department of Physics and Institute for Functional Nanomaterials, P.O. Box 70377, University of Puerto Rico, San Juan, PR 00936-8377 USA; 2 Department of Physics, The Pennsylvania State University, University Park, Pennsylvania, 16802 USA; 3 School of Physics, University of St. Andrews, St. Andrews, KY19 9SS UK

**Keywords:** Materials science, Nanoscience and technology

## Abstract

The discovery of single-phase multiferroic materials and the understanding of coupling mechanisms between their spin and polarization is important from the point of view of next generation logic and memory devices. Herein we report the fabrication, dielectric, ferroelectric, piezo-response force microscopy, and magnetization measurements of Pd-substituted room-temperature magnetoelectric multiferroic PbPd_0.3_Ti_0.7_O_3_ (PbPdT) thin films. Highly oriented PbPdT thin films were deposited on {(LaAlO_3_)_0.3_(Sr_2_AlTaO_6_)_0.7_} (LSAT) substrates in oxygen atmosphere using pulsed laser deposition technique. X-ray diffraction studies revealed that the films had tetragonal phase with (001) orientation. Surface morphology studies using atomic force and scanning electron microscopy suggest a smooth and homogeneous distribution of grains on the film surface with roughness ~2 nm. A large dielectric constant of ~1700 and a low-loss tangent value of ~0.3 at 10 kHz were obtained at room temperature. Temperature dependent dielectric measurements carried out on Pt/PbPdT/La_0.7_Sr_0.3_MnO_3_ (LSMO) metal-dielectric-metal capacitors suggest a ferroelectric to paraelectric transition above 670 K. The measured polarization hysteresis loops at room temperature were attributed to its ferroelectric behavior. From a Tauc plot of (αhν)^2^ versus energy, the direct band gap E_g_ of PbPdT thin films was calculated as 3 eV. Ferroelectric piezoelectric nature of the films was confirmed from a strong domain switching response revealed from piezo-response force microscopy. A well-saturated magnetization *M*-*H* loop with remanent magnetization of 3.5 emu/cm^3^ was observed at room temperature, and it retains ferromagnetic ordering in the temperature range 5–395 K. Origin of the magnetization could be traced to the mixed oxidation states of Pd^2+^/Pd^4+^ dispersed in polar PbTiO_3_ matrix, as revealed by our x-ray photoelectron spectroscopic results. These results suggest that PbPdT thin films are multiferroic (ferroelectric-ferromagnetic) at room temperature.

## Introduction

**Figure 1:**
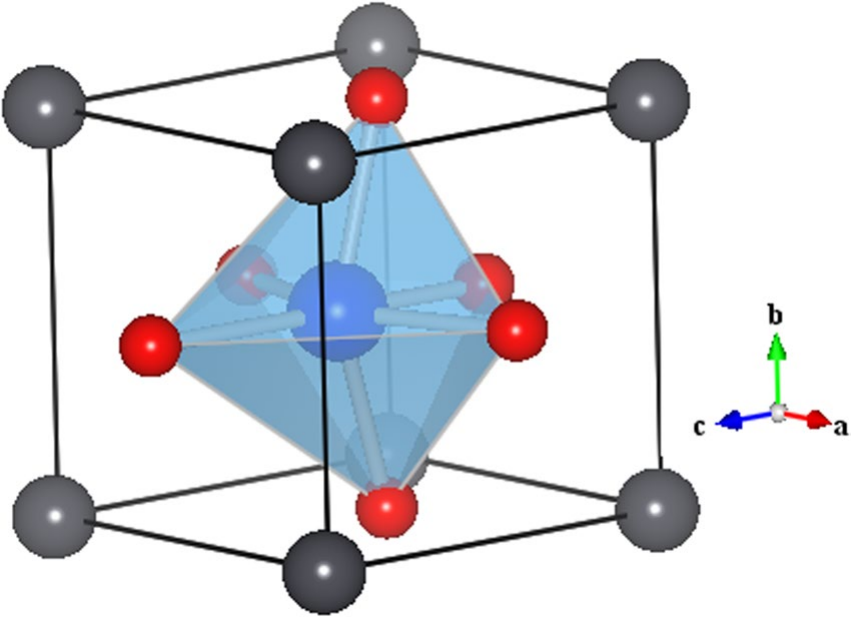
*AB*O_3_ type structure of PbTiO_3_: drawn using VESTA software^47^.

Multiferroic materials with coupled electric and magnetic ordering in a single phase are of research interest due to their fascinating physics and potential for multifunctional device applications, such as speed writing and non-destructive data storage^1–4^. Only a limited number of single phase multiferroics are available due to the chemical incompatibility between magnetic and ferroelectric order parameters^2^. The most well-known examples are: BiFeO_3_, Pb(Fe_0.5_Nb_0.5_)O_3_, YMnO_3_, TbMnO_3_, GaFeO_3_^5–11^. However, a majority of these materials exhibit multiferroicity at cryogenic temperature. Materials exhibiting multiferroicity at room temperature are important for device applications, but only a few have been discovered so far, the most celebrated examples being BiFeO_3_^10,12^, epitaxial thin films of GaFeO_3_^13^ and Bi_2_FeCrO_6_^14–16^. During last decade, extensive studies have been carried out to investigate BiFeO_3_^10–12,17^ that exhibits both ferroics ordering above room temperature with ferroelectric to paraelectric phase transition temperature T_c_ ~ 1143 K and antiferromagnetic to paramagnetic phase transition Néel temperature T_N_ ~ 643 K, to understand the origin and mechanism of its multiferroicity. Pd-substituted PbTiO_3_-based oxides are new promising room temperature multiferroic materials due to coexistence of ferroelectric and magnetization ordering at and above room temperature. Lead titanate (PbTiO_3_) is a classical ferroelectric at room temperature, belonging to the perovskite family with the general formula *AB*O_3_ (Fig. 1). The sublattice *A* is occupied by non-magnetic Pb^2+^ (6P^0^, S = 0) and the *B*-site is occupied by non-magnetic Ti^4+^ (3d^0^, S = 0) ions. It undergoes a first-order displacive phase transition from ambient tetragonal to high temperature cubic phase at T_c_ ~ 763 K^18^. However, recent density functional theoretical (DFT) studies^19^ have predicted ferromagnetism in Pd-substituted PbTiO_3_. They revealed that the isoelectronic substitutions of Pd^2+^ at the *A*-site (Pb^2+^ ion) and Pd^4+^ cation at the *B*-site (Ti^4+^ ion) do not establish magnetic ordering. However, replacement of Pb^2+^ by Pd^4+^ ion produces magnetism in PbTiO_3_, and this is argued to be due to donation of two electrons by the substituted Pd^4+^ cation to the defect states in the band gap lying between the conduction and valence bands^10,19^. Remarkably, Pd cation (Pd^2+^, Pd^4+^) substitution on Pb site is complicated as the radii of these two cations are not similar and latter is oversized for Pd cations. Earlier studies suggested that Pd atom is as such non-magnetic (4d^10^, S = 0)^20^; however, under the influence of external electric field and/or strain, Pd cation can become ferromagnetic and its magnetic ordering can be electrically tunable^21–24^. A single phase of 30% Pd-substituted Pb(Zr_0.20_Ti_0.80_)_0.70_Pd_0.30_O_3−δ_ (PZTP30) ceramic was successfully prepared by solid state reaction method, that is reported to be room temperature multiferroic^14^. It exhibited a weak ferromagnetism, improved ferroelectricity, and importantly showed strong magneto-electric (ME) coupling. Very recently, Pd-doping of up to 30% in ferroelectric PbTiO_3_ was realized experimentally that established its magnetoelectric multiferroicity at room temperature, superior to BiFeO_3_^25^. The magnetism in the ceramic sample was ascertained from the presence of Pd^4+^ chemical state from x-ray photo electron spectroscopy (XPS). However, thin films fabrication of this intriguing multiferroic and investigation of their polarization and magnetization (multiferroic order parameters) are yet to be carried out. Multifunctional thin films are preferable for device applications such as in highly sensitive actuators and sensors, multistate memories, and nanoelectronics^10,26–29^, and they are also fascinating for their rich physics. Pulsed laser deposition technique is a sophisticated thin film growth technique often useful for the fabrication of high purity thin films with nanoscale precision^30–33^ and is preferable for deposition of high quality films including epitaxial films on appropriate substrates.

We report the fabrication of PbPd_0.3_Ti_0.7_O_3_ (PbPdT) thin films using pulsed laser deposition technique and investigations of their structural, dielectric, ferroelectric, and magnetic ordering behavior with temperature. We have fabricated Pt/PbPdT/La_0.7_Sr_0.3_MnO_3_(LSMO)/{(LaAlO_3_)_0.3_(Sr_2_AlTaO_6_)_0.7_} (LSAT) heterostructures for electrical measurements, and studied the magnetization of PbPdT thin films (of similar thickness) grown on LSAT (001) substrates in order to avoid any magnetization contribution from La_0.7_Sr_0.3_MnO_3_ (LSMO). These films had *c*-axis orientation. Study of the role of chemical substitution of Pd ions on the magnetic properties of the films revealed that the PbPdT films exhibited well defined *M*-*H* hysteresis loops up to 395 K and retained ferromagnetic ordering from 5 to 395 K, the highest temperature in the present study. X-ray photo electron spectroscopic measurements revealed mixed oxidation states of Pd^2+^ and Pd^4+^ in Pd-substituted polar PbTiO_3_ matrix and fulfilled the requirement to realize ferromagnetism in PbPdT films. Dielectric studies on Pt/PbPdT/LSMO metal-insulator-metal (MIM) capacitors suggest that the ferroelectric to paraelectric phase transition temperature is above 670 K. From the Tauc plot analysis of absorption spectra using (αhν)^2^ versus energy, the direct band gap E_g_ of PbPdT thin films is estimated as 3 eV. The ferroelectric piezoelectric nature of the films is evident from the strong domain switching response obtained from the piezoresponse force microscopy (PFM) studies involving color contrast phase and amplitude images. Furthermore, magnetocapacitance effect was noticed in our thin films.

## Results and Discussion

**Figure 2:**
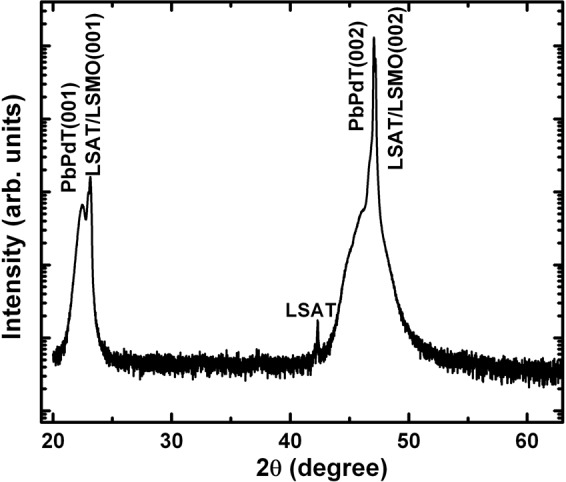
X-ray diffractogram measured in *θ*-2*θ* geometry for a PbPd_0.3_Ti_0.7_O_3_ film grown on LSMO coated LSAT (001) substrate. The reflection peaks are also indexed for PbPd_0.3_Ti_0.7_O_3_.

**Figure 3:**
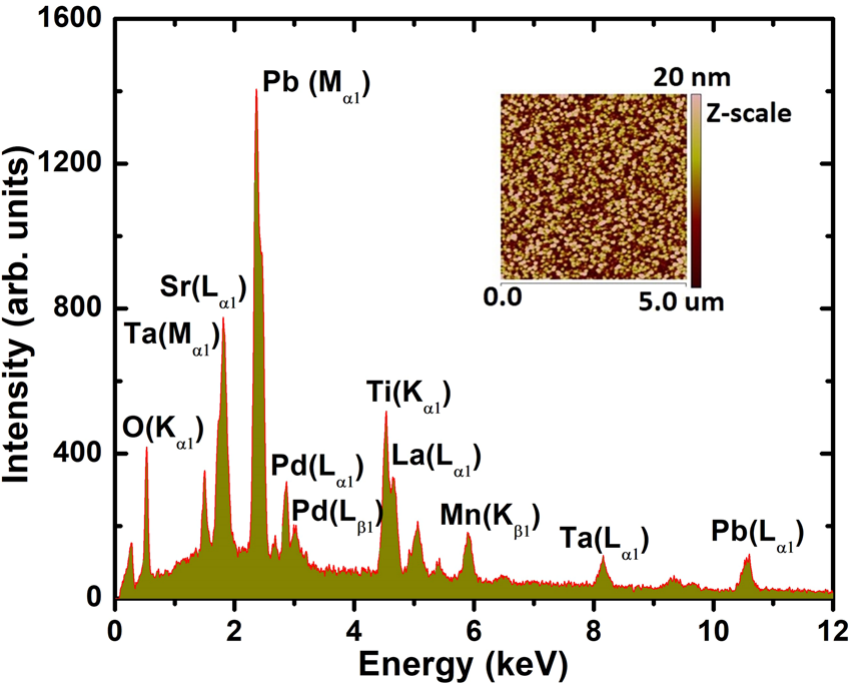
EDS spectrum for a PbPd_0.3_Ti_0.7_O_3_ film shows the presence of all constituent elements at their respective characteristic energy level (Inset: AFM image; 20 nm in Z-scale of a PbPdT film).

**Figure 4:**
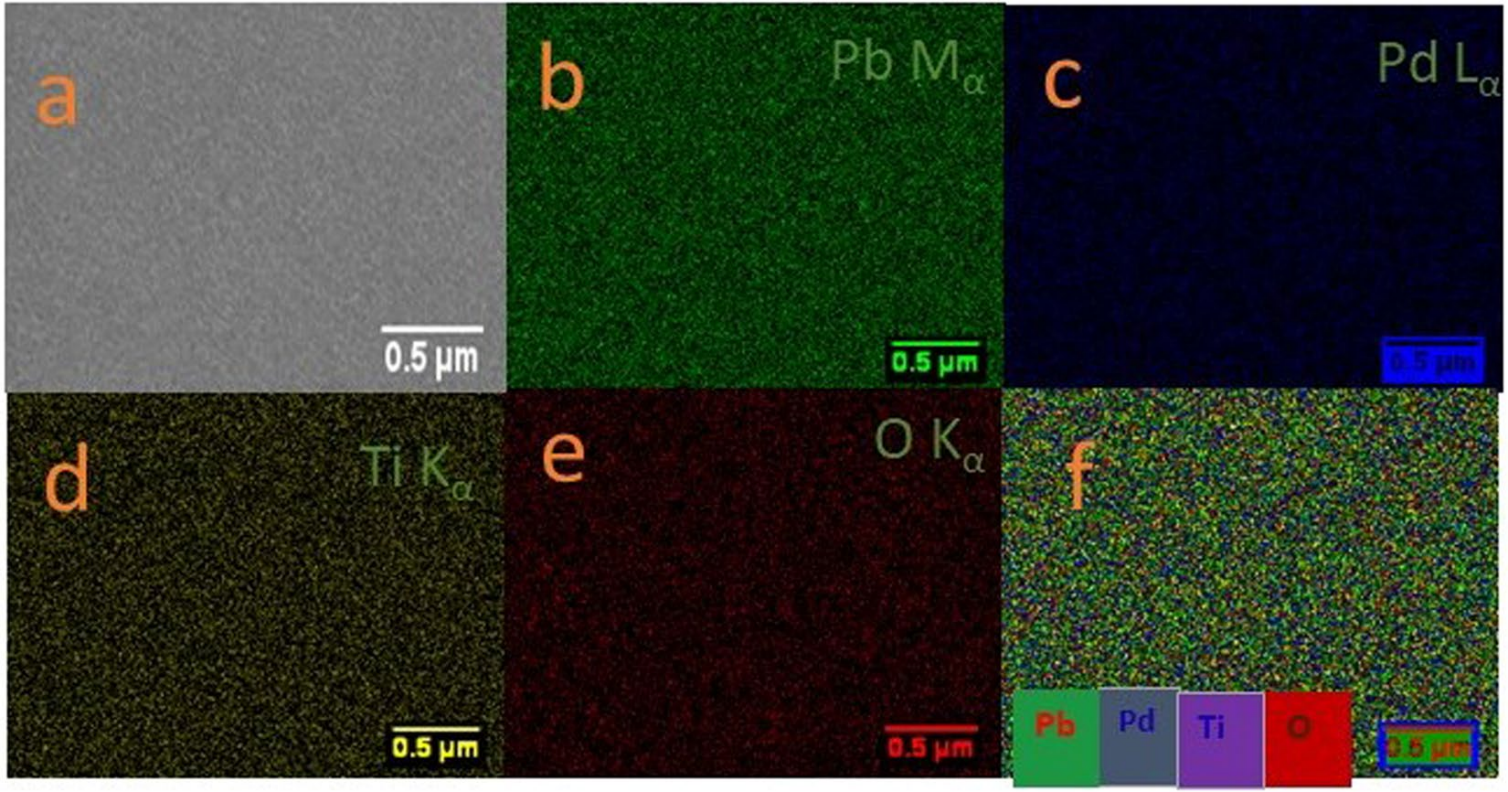
(**a**) SEM micrograph of a PbPd_0.3_Ti_0.7_O_3_ film. Distribution maps of elements (**b**) Pb, (**c**) Pd, (**d**) Ti, and (**e**) O, and (**f**) sum total elemental mapping of the surface area.

The PLD deposition parameters were optimized for the growth of PbPdT thin films as summarized in Table 1. The high-resolution x-ray diffraction pattern of one of the PbPdT thin films deposited on LSMO buffer layer coated on LSAT substrate is shown in Fig. 2. As can be seen, in the *θ*-2*θ* scan, the reflections corresponding to PbPdT, LSMO and LSAT substrate were noticed in the 2*θ* range from 20°–60°. In addition, absence of any impurity peaks originating from other secondary phases indicated that the grown PbPdT thin film is phase pure and stabilized in a single perovskite phase The thin films were stabilized in the tetragonal phase (*P4mm*), as in pristine PbTiO_3_ (JCPDF # 742495), and these were oriented with their (001) plane parallel to the substrate surface along with their out-of-plane lattice parameter *c* as 3.958 Å. Lattice mismatch between the thin film and the substrate induces a structural strain, which can be estimated using the relation^33^, η = (*a*_substrate_ − *a*_film_/*a*_film_) × 100. The LSAT and LSMO stabilized in cubic perovskite phase with their lattice parameters *a*_substrate_ = 3.868 Å^34^ and *a*_LSMO_ = 3.871 Å^34^, respectively. However, the in-plane lattice parameters (*a* and *b*) of the films can not be extracted from the present diffraction result (Fig. 2). In order to obtain these lattice parameters, in-plane x-ray diffraction and/or Reciprocal Space Mapping measurements are required. In the absence of these extra experimental results, the in-plane lattice strain in the films could not be calculated. The thickness of the films, estimated using a Profilometer, was found to be ~300 nm. The presence of the constituent elements, such as Pb, Pd, Ti, and O in the films were inferred from an energy dispersive x-ray spectrum (EDS) (Fig. 3) excited using an electron beam of kinetic energy 20 keV. X-ray emission lines corresponding to O K_α1_ 0.53 keV, Pb M_α1_ 2.34 keV, Pd L_α1_ 2.88 keV, Pd L_β1_ 3.01 keV, Ti K_α1_ 4.53 keV and Pb L_α1_ 10.58 keV exactly match with the available theoretical characteristic x-ray emission lines. The surface topography of the thin films was measured using atomic force microscopy (AFM) (Fig. 3: inset) from the surface of the thin films indicating a smooth surface of the films with its root mean square roughness (R_Q_) of ~2 nm. The surface morphology of the films using scanning electron microscopy (SEM) is shown in Fig. 4a. It suggests that the thin films are smooth and dense with close packed crystal grains. A homogeneous distribution of these chemical elements was ascertained via SEM-based elemental mapping profiles (Fig. 4b–f), scanned over a large surface area of the films.

**Table 1:** Growth parameters for PbPd_0.3_Ti_0.7_O_3_ thin films deposited using pulsed laser deposition technique.

Substrate	{(LaAlO_3_)_0.3_(Sr_2_AlTaO_6_)_0.7_} (001)
Target	Pb_0.7_Pd_0.3_TiO_3_
Target diameter	0.025 m
Substrate target distance	0.05 m
Growth temperature	923 K
Base vacuum	~10^−6^ Torr
O_2_ partial pressure	200 mTorr
Laser source	excimer gas (λ = 248 nm, KrF)
Laser energy used	250 mJ
Pulse repetition rate	10 Hz
Total number of shots	3000
Post deposition *ex*-*situ* annealing	923 K for 0.5 hour in O_2_ atmosphere

**Figure 5:**
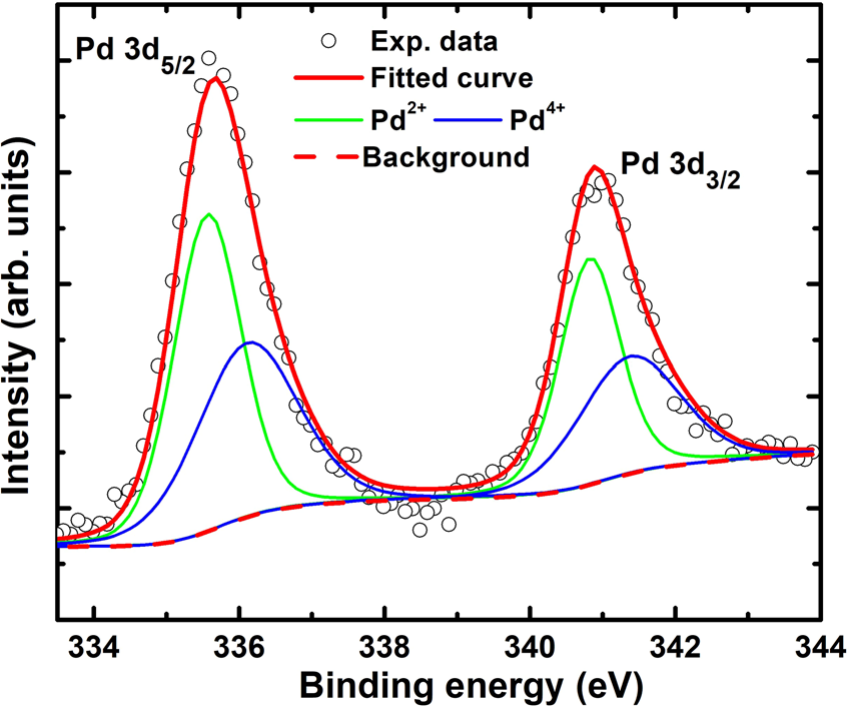
X-ray photoelectron spectroscopic analysis for the Pd element. The individual deconvoluted peaks of Pd 3d_5/2_ and Pd 3d_3/2_ doublet of Pd 3d spectrum are also shown. (CasaXPS, Version 2.3.22, http://www.casaxps.com/).

As mentioned earlier, it was argued from DFT studies^19^ as well as other earlier experimental reports on Pd-based ceramics^19,25^ that the origin of magnetism in PbPdT is due to the existence of Pd^4+^ cationic state at Pb^2+^ site (*A*-site). Therefore, it is imperative to examine the valence state of palladium (Pd) using x-ray photoelectron spectroscopy. From the survey spectra, the presence of all constituent elements, as inferred from EDS spectra, were confirmed from their respective binding energy matching with the standard theoretical values. The high resolution XPS spectrum of Pd consists of two components due to spin-orbit coupling effect (Fig. 5). The spin-orbit doublets of Pd were found to be asymmetric with peak centers at Pd 3d_5/2_ = 335.65 eV and Pd 3d_3/2_ = 340.91 eV, suggesting the presence of Pd^4+^ and Pd^2+^ valence states. These doublets were deconvoluted using the Casa XPS software and by choosing a suitable background. The deconvolution of Pd 3d_5/2_ peak yielded Pd^2+^, and Pd^4+^ states at 335.56 eV, and 336.11 eV, respectively. Similarly, the Pd 3d_3/2_ peak can be resolved into Pd^2+^ state at 340.82 eV, and Pd^4+^ state at 341.37 eV (Fig. 5). The effective area ratio Pd^4+^:Pd^2+^ was calculated to be 0.83: 1. The smaller value of Pd^4+^ states can be explained by oxygen loss from the surface, that turns some Pd^4+^ into Pd^2+^ states. The observed magnetism in our PbPdT thin films is due to mixed oxidation states of Pd that essentially fulfills the requirement to realize the ferromagnetism in Pd-based perovskites^19,25^.

**Figure 6:**
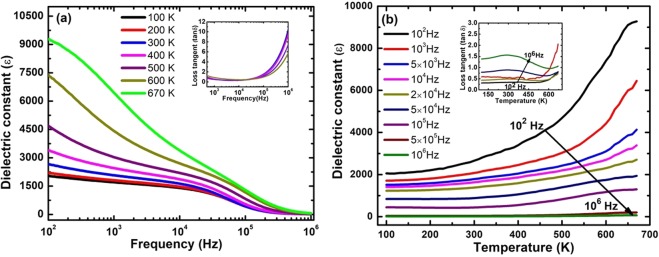
(**a**) Frequency dependencies of dielectric constant (ɛ) of PbPd_0.3_Ti_0.7_O_3_ thin films measured at different temperatures (Inset: dissipation factor (tan δ) with frequencies at different temperatures), (**b**) Temperature dependent dielectric constant (ɛ) of PbPd_0.3_Ti_0.7_O_3_ thin films. (Inset: dissipation factors (tan δ) with temperatures).

The dielectric constant (ɛ) and the loss tangent (tan δ) of PbPdT thin films were measured in the frequency range 10^2^–10^6^ Hz and temperature between 100–670 K (Fig. 6(a)). The room temperature dielectric constant and dielectric loss were found to be ~1700 and ~0.3, respectively, measured at 10 kHz. The dielectric constant ɛ was found to be higher than the reported values for PZTP30 (~300) and PbPdT sintered bulk samples (~500). One can notice a nominal decrease in dielectric constant and almost constant loss tangent values with increasing frequency up to 10^5^ Hz. However, a substantial decrease in the dielectric constant and a rise in dielectric loss were observed above 10^5^ Hz frequency. The reduction in ɛ is expected since the dielectric constant of PbPdT thin films has contributions from intrinsic factors related to the lattice, and extrinsic contributions involving grain boundary and interface. In particular, the intrinsic contribution is dependent upon the grain size, film orientation, and strain on the films. However, the interface between the PbPdT film and the bottom LSMO electrode acts as a pinning center that contributes extrinsically^34^ to the dielectric constant. It perturbs the domain wall motion, and consequently leads to a reduction in the dielectric constant. Such a reduction of dielectric constant at high frequencies was reported in Pb(Zr_0.52_Ti_0.48_)O_3_/LSMO thin films^34^, and it was argued to arise due to interfacial contributions. A close inspection of the dielectric behavior of the reported LSMO films^35^, and the present PbPdT/LSMO films at high frequencies indicate a similar decreasing trend suggesting that the dielectric response of our thin film capacitors (>10^5^ Hz) could be mainly from the bottom layer (LSMO). The loss tangent values become larger at higher frequency which can be attributed to the semiconducting nature of the bottom layer^36^, and also to the presence of disorder or defects in the films^37,38^. The present dielectric behavior is similar to those reported in other perovskite ferroelectrics, such as Pb(Zr_0.53_Ti_0.47_)_0.9_Sc_0.1_O_3_^27^, Pb(Zr_0.2_Ti_0.8_)_0.3_O_3-δ_^36^. At high temperatures (≥400 K), a substantial drop in the dielectric constant at high frequencies (>10^5^ Hz) is observed due to thermal activation of hopping motion of oxygen vacancies. As in several perovskite oxides^37,39^, ionic conductivity in PbPdT films is expected to increase due to increase in movement of oxygen vacancies with thermal energy. The temperature dependent behavior of dielectric constant and loss tangent in the frequency range 100 Hz −1 MHz is shown in Fig. 6(b). The dielectric constant was found to be almost constant up to 400 K, followed by a drastic increase above this temperature. A sharp rise in dielectric constant above 670 K could indicate that the system is approaching the ferroelectric-paraelectric phase transition temperature. Using our temperature controller MMR K-20, the heater power allows us to reach only up to 670 K, therefore the actual dielectric maximum that is likely to be above this temperature (≥670 K), could not be observed. Indeed the ferroelectric Curie temperature T_c_ in PbPdT ceramics is reported to be 740 K^25^. Pd cation is larger in size compared to Ti cation, therefore upon substituting such a large size Pd cation at the Ti-site in PbTiO_3_, the unit cell volume increases. Hence the transition temperature T_c_ is expected to reduce compared to the pristine PbTiO_3_ films (T_c_ ~ 823 K)^40^. The rise of loss tangent at higher temperatures is due to space charge polarization contribution.

**Figure 7:**
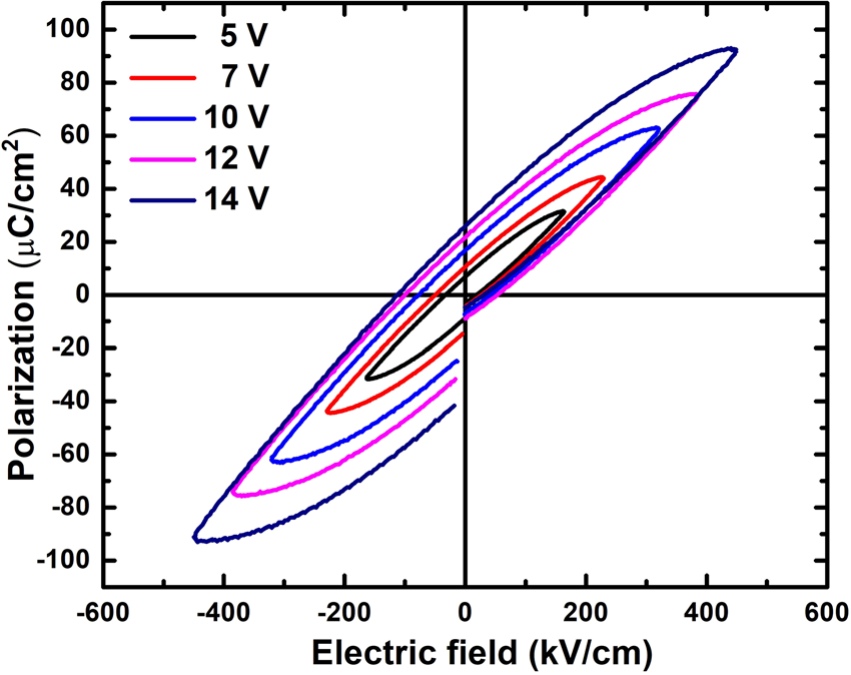
*P*-*E* hysteresis loops of LSMO/PbPd_0.3_Ti_0.7_O_3_/Pt thin film capacitors measured at various applied fields at frequency of 10 kHz.

*P*-*E* hysteresis loops of PbPdT thin film capacitors measured at various applied fields at 10 kHz frequency at room temperature are shown in Fig. 7. With increasing electric field from 0 to ~500 kVcm^−1^, both the polarization maximum P_max_ and the remanent polarization P_r_ increase and are likely to reach a saturation value at higher electric fields than the present field range. A possible reason for non-saturation of *P*-*E* loop is that the conductivity originates from oxygen vacancies and other ionic conduction processes^25^. In fact, several Pd-based perovskites are reported to possess large conductivity due to Pd cation effect^25,41,42^. The current-voltage (*I*-*V*) characteristics of thin film oxides support significant conductivity (leakage current) through the films with bias voltage (discussed later). The *P*-*E* loop of our thin films showed a maximum polarization of 92 µCcm^−2^ and a remanent polarization of 27 µCcm^−2^, larger than those measured in other Pb-based bulk counterparts^19^. The coercive field E_c_ was obtained as 55 kVcm^−1^, which is also larger compared to the Pd-based ceramics (6.5 kVcm^−1^)^19^. In thin films, defects such as oxygen vacancies, interface layers and several other impurities generate an internal electric field^43,44^. The observed discontinuity in the hysteresis loops is a result of the polarization relaxation due to this field. Such discontinuities in *P*-*E* loops were also reported in other ferroelectric materials, e.g. (Ba_0.955_Ca_0.045_)(Zr_0.17_Ti_0.83_)O_3_^45^ and Pb-based ferroelectric films^43,44^. Since the structure has a polar axis, a definite proof of ferroelectricity is later confirmed from our piezoresponse force microscopy experiments.

**Figure 8:**
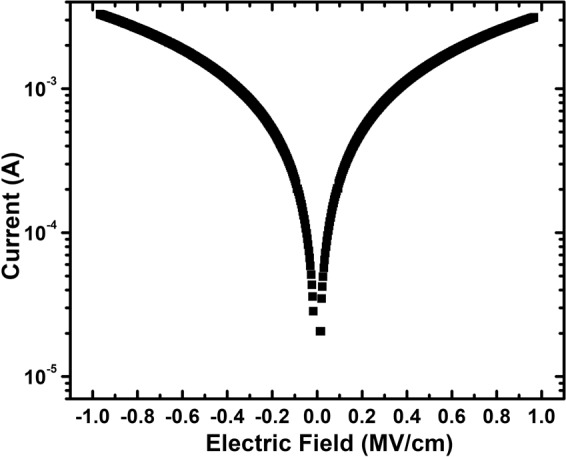
Variation of leakage current with applied electric field for the PbPdT films.

Figure 8 shows the typical leakage current conduction behavior i.e. current-voltage *(I*-*V*) characteristic curve of the PbPdT thin films, measured with a voltage step of 0.1 V and elapsed time of 0.5 s at each voltage step. Two distinct regions are noticed in the *I*-*V* curve: below 0.11 MV/cm, the current increases linearly with applied electric field suggesting an Ohmic conduction behavior, and above 0.11 MV/cm, the current increases exponentially with increasing electric field, which is reminiscent of Schottky or Poole-Frankel emission type conduction processes^45^. A significant leakage current (>0.001 A) through the films is seen above 0.4 MV/cm, and it can be attributed to conductivity that originates from oxygen vacancies, and interface limited conduction processes^25^. Similar conduction behavior was observed in PLD based (Ba_0.955_Ca_0.045_)(Zr_0.17_Ti_0.83_)O_3_ thin film capacitors, exhibiting a large leakage current density above an applied field of 0.7 MV/cm^45^.

**Figure 9:**
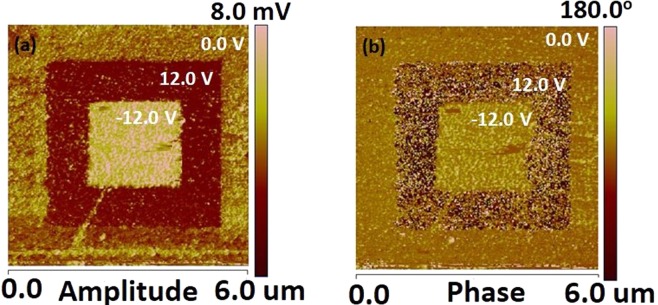
Piezoresponse force microscopy: (**a**) amplitude, and (**b**) phase images of PbPdT/LSMO/LSAT thin films of thickness 300 nm. The large square area of (6 × 6) µm^2^ and central square area of (4 × 4) µm^2^ of the thin films were polled with +12 V and −12 V bias dc voltages, respectively.

To study the ferroelectricity at nanoscale, PFM measurements were carried out on the surfaces of PbPdT films. Conducting Pt/Ir cantilever tip was used as PFM tip and it acted as the top electrode, and LSMO as the bottom electrode was grounded to obtain the piezo response images. A strong domain switching response was inferred from the amplitude and phase images (Fig. 9). A large square area of (6 × 6) µm^2^ and other central square area of (4 × 4) µm^2^ of the thin films were polled with +12 V and −12 V bias dc voltages, respectively. The domains with opposite polarization were distinguished with dark and bright contrast in the image. Two color contrast polling regions are clearly seen indicating the polar switching behavior in the PbPdT films in nanoscale, and the ferroelectric piezoelectric nature of the films is confirmed as that inferred from our dielectric and ferroelectric studies.

**Figure 10:**
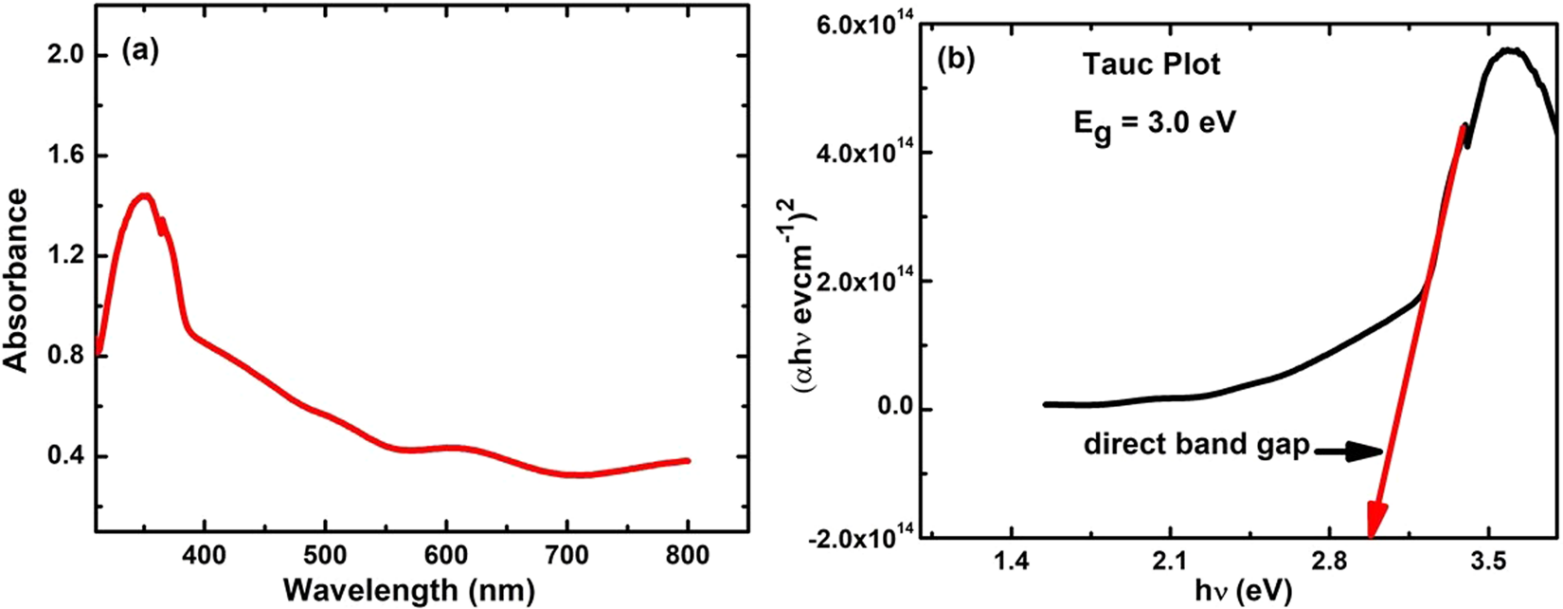
(**a**) UV-Visible absorption spectrum between absorbance and wavelength, (**b**) Optical band gap E_g_ of PbPd_0.3_Ti_0.7_O_3_ thin films using UV-Visible absorbance spectrum using Tauc plot.

The optical absorption spectra of the thin films were measured employing a UV-2400PC spectrophotometer in the wavelength range of 300–800 nm. The substrate contribution was excluded from the spectrum by using a similar ITO-coated glass substrate as a reference. The UV-visible spectrum (Fig. 10(a)) of the PbPdT thin films show an absorption edge at 390 nm. From the Tauc plot of (αhν)^2^ versus energy (Fig. 10(b)), the direct band gap E_g_ was calculated as 3 eV, which is found to be less than that reported for PbTiO_3_ thin films^18,46^. Thus, a reduction in band gap upon Pd substitution is apparent.

**Figure 11:**
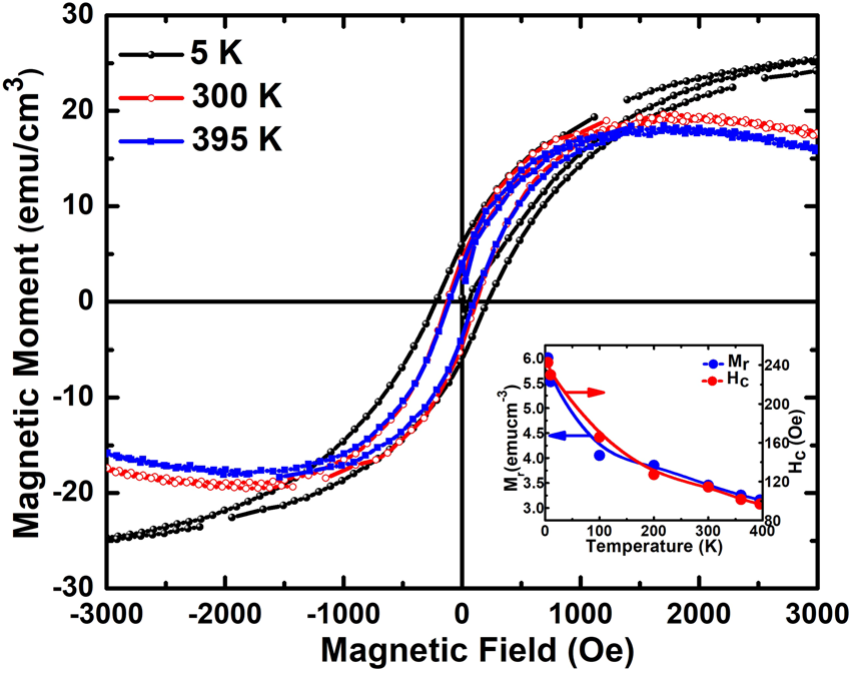
*M*-*H* curves measured at 5 K, 300 K and 395 K. Inset: the temperature dependence of M_r_ and H_c_ (lower panel).

**Figure 12:**
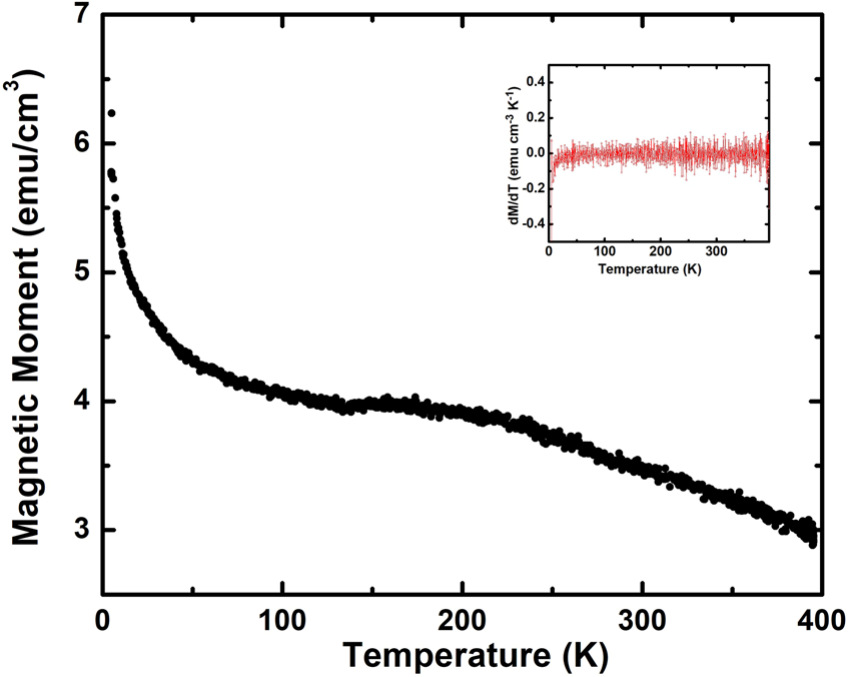
Temperature evolution of the remanent magnetization created at 5 K with applied saturation field of 2 T, for PbPd_0.3_Ti_0.7_O_3_ films. The inset figure shows the derivative (d*M*/d*T*) of magnetization *M* with temperature *T*.

Figure 11 shows the magnetization hysteresis curves for the PbPdT films at various temperatures. The films exhibited clear magnetic hysteresis loops indicating ferromagnetism in the films. At the lowest temperature of 5 K, the saturation magnetization (M_s_) and the coercive field (H_c_) of the films were 6 emu-cm^−3^ and 242.7 Oe, respectively. DFT calculations suggest that the substitution of non-isoelectric Pd^4+^ cation at the Pb^2+^ site of PbTiO_3_ generates two electrons, which occupy the induced defect states in the electronic band gap of PbTiO_3_. These electrons are believed to produce magnetism^19^. Our XPS studies confirmed the presence of mixed oxidation states of Pd ions (Pd^2+^, Pd^4+^) that fulfilled the condition for magnetization in the films. To experimentally demonstrate that the Pd^4+^ is at the A-site, experimental works providing local bonding coordination are required, which we are planning to carry out separately. The temperature dependence of the magnetization was studied by carrying out measurements of magnetization hysteresis loops (*M*-*H* curve) at different temperatures (Fig. 11). A plot of the coercive field H_c_ and the remanent magnetization M_r_ of PbPdT films as a function of temperature is inset in Fig. 11. Upon increasing temperature, M_r_ and H_c_ values of the hysteresis loops decrease. These H_c_ and M_r_ values obtained at different temperatures are presented in Table 2. The H_c_ value at 300 K (114 Oe) is about 2 times less than that observed at 5 K (242 Oe). Similarly, the M_r_ value at 300 K (3.4 emucm^−3^) reduces by nearly half compared to that at 5 K (6 emucm^−3^). The appearance of magnetic hysteresis loop in the films at 395 K indicates that the films are ferromagnetic with a Curie temperature above 395 K. The small discontinuity in hysteresis loop could be due to a few missing data points in our measurements due to the lock-in amplifier (or other electronics) changing the range of measurements in the PPMS system. To examine if there are any changes in the magnetic ordering of the films, we have investigated the temperature evolution of magnetization from 5 to 395 K. A saturation field of 2 T was applied at 5 K, and the field was turned off thereafter. In the absence of the field (*H* = 0), thermal evolution of magnetization was measured up to 395 K (Fig. 12). A gradual reduction of magnetization with increasing temperature is noticed. The rate of change of magnetization with temperature (d*M*/d*T*) that provides information about any changes in the magnetic ordering is inset in Fig. 12. In principle, any change in magnetic ordering would result in a significant change in the magnetization curve. Since d*M*/d*T* remains constant, it is evident that there is no change in magnetic ordering in the films up to 395 K. Thus, the films are ferromagnetic throughout the temperature range of the present studies and the magnetic Curie temperature is above 395 K.

**Table 2:** Remanent magnetization M_r_ and coercive field *H*_c_ at different temperatures (5–395 K) for PbPd_0.3_Ti_0.7_O_3_ films.

Temperature (K)	M_r_ (emucm^−3^)	H_c_ (Oe)
5	6.007	242.729
10	5.529	229.906
100	4.052	165.788
200	3.853	127.318
300	3.455	114.495
360	3.256	101.671
395	3.162	96.862

## Conclusions

(001)-oriented PbPd_0.3_Ti_0.7_O_3_ thin films were grown on LSAT single crystal substrates using pulsed laser deposition technique. X-ray diffraction analysis indicates that the grown thin films are phase pure and stabilized in tetragonal phase. Temperature dependent dielectric studies on Pt/PbPdT/LSMO metal-insulator-metal capacitors suggest that the ferroelectric Curie temperature is above 670 K. The polarization hysteresis loops at room temperature were attributed to its ferroelectric behavior. The *P*-*E* loop of our thin films showed a maximum of 92 µCcm^−2^ polarization with remanent polarization P_r_ of 27 µC cm^−2^ at an applied electric field of 500 kVcm^−1^. A saturated magnetization *M*-*H* loop with remanent magnetization M_r_ of 3.5 emu/cm^3^ was observed at room temperature and the film retains ferromagnetic ordering up to as high as 395 K. X-ray photoelectron spectroscopic results confirmed the mixed chemical states of the Pd 3d state with Pd^4+^: Pd^2+^ of 0.83: 1. Magnetization in the PbPdT films is due to the existence of mixed oxidation states of Pd^2+^/Pd^4+^ and this fulfills the predicted requirement to realize ferromagnetism in PbPdT films. From the Tauc plot of (αhν)^2^ versus energy, the direct band gap of PbPdT thin films was estimated as 3 eV. Ferroelectric piezoelectric nature of the films was confirmed from a strong domain switching response revealed from our piezoresponse force microscopy studies on the phase and amplitude contrast images. The experimental results revealed that the PbPdT thin films are multiferroic (ferroelectric-ferromagnetic) at room temperature and have potential for device applications in non-volatile memories, transducers, and actuators.

## Methods

PbPdT, and PbPdT/LSMO thin films were grown on {(LaAlO_3_)_0.3_(Sr_2_AlTaO_6_)_0.7_} (LSAT) (001) substrates in an oxygen atmosphere using a KrF gas excimer laser operated at λ = 248 nm with a pulse repetition rate of 10 Hz using PLD technique. PbPdT target with a stochiometric molecular formula was prepared employing the solid-state reaction method following the procedure reported earlier^25^. The PLD deposition parameters were optimized for the growth of PbPdT thin films as summarized in Table 1. Using an XP-200 Profilometer the thickness of the films was measured to be ~300 nm. Elemental compositions and their mapping distributions were studied using Energy Dispersive X-ray Spectroscopy (EDS) employing a scanning electron microscope (JEL JSM-6480LV). Atomic Force Microscopy (AFM-Veeco) was operated in contact mode to study the surface topography of thin films and their surface roughness. The crystallographic phase and orientation of these grown thin films were confirmed from the analysis of high-resolution XRD data measured using a Smartlab x-ray diffractometer employing a Cu-Kα radiation (λ = 1.5405 Å). The chemical states of the constituent elements were studied using x-ray photo emission spectroscopy (XPS). For electrical measurements, PbPdT capacitors sandwiched between a conducting bottom LSMO layer and a top platinum (Pt) electrode were fabricated. Pt electrodes were fabricated by D.C. sputtering technique using a metal shadow mask of area 10^−8^ m^2^ followed by annealing at 620 K in an oxygen environment to recover any defects generated during sputtering. The temperature dependent dielectric properties such as capacitance and dielectric loss tangent (tan δ) were measured at several frequencies (100 Hz to 1 MHz) in the temperature range of 100–670 K by employing a programmable temperature controller (MMR K-20) with a temperature stability of ±1 K and using an impedance analyzer (HP 4294 A). Ferroelectric hysteresis loops (*P*-*E* curves) were measured at ambient temperature using a hysteresis loop tester (radiant Technologies RT6000 HVS). Magnetization measurements were carried out using a physical properties measurement system (PPMS, Quantum design), operated in the VSM module in the temperature range of 5–395 K. Piezoresponse force microscopy (PFM) studies on thin films were carried out at ambient temperature using a Multimode Nanoscope V (Veeco Instruments). The used conductive tips were coated with Pt/Ir having a resonance frequency of 140 kHz and force constant of 4.5 Nm^−1^. During PFM measurements, the driving applied voltages on the film surface was ±12 V to monitor the material’s polarization switching and amplitude.

## Data Availability

The data sets are available from the corresponding author on reasonable request.
